# Trends in Ultraprocessed Food Consumption Among Korean Children and Adolescents, 2007 to 2024

**DOI:** 10.1001/jamanetworkopen.2026.5528

**Published:** 2026-04-07

**Authors:** Sukyoung Jung, Eunice Hong Lim Lee, Jee Young Kim, Sohyun Park, Jung Eun Lee

**Affiliations:** 1 Department of Healthcare Policy Research, Korea Institute for Health and Social Affairs, Sejong, Korea; 2 Jeju Jinsan Company, Seogwipo, Korea; 3 Department of Food Science and Nutrition, Hallym University, Chuncheon, Korea; 4 The Korean Institute of Nutrition, Hallym University, Chuncheon, Korea; 5 Department of Food and Nutrition, College of Human Ecology, Seoul National University, Seoul, Korea; 6 Research Institute of Human Ecology, College of Human Ecology, Seoul National University, Seoul, Korea

## Abstract

**Importance:**

The prevalence of childhood obesity has tripled between 1999 and 2021, and the greatest increases have been observed in Asia. Ultraprocessed foods are considered a major risk factor for obesity; however, trends in ultraprocessed food consumption among Korean youths remain understudied.

**Objective:**

To examine 18-year trends in ultraprocessed food consumption stratified by obesity and demographic characteristics among Korean youths.

**Design, Setting, and Participants:**

This population-based cross-sectional study used 24-hour dietary recall data from 6 cycles of the Korea National Health and Nutrition Examination Survey from 2007 to 2024. Participants included Korean youths aged 1 to 18 years.

**Exposure:**

Survey cycles.

**Main Outcomes and Measures:**

The percentage of energy intake from ultraprocessed foods based on the Nova food classification system was estimated across survey cycles.

**Results:**

Among the 24 518 youths included, the weighted mean (SD) age of participants was 10.2 (5.1) years, of whom 12 797 (52.2%) were boys. From the 2007-2009 to 2022-2024 cycles, the percentage of energy from ultraprocessed foods increased from 24.6% (SE, 0.5%) to 33.0% (SE, 0.5%) (*P* < .001 for trend), while the percentage of energy from unprocessed or minimally processed foods decreased from 64.8% (SE, 0.5%) to 51.8% (SE, 0.4%) (*P* < .001 for trend). There was a greater increase in ultraprocessed food consumption among school-aged children (from 22.9% [SE, 0.6%] to 34.0% [SE, 0.6%]) and adolescents (from 28.7% [SE, 0.8%] to 36.9% [SE, 0.7%]) than among preschool-aged children (from 23.5% [SE, 0.6%] to 26.4% [SE, 0.9%]) and among youths without obesity (from 24.7% [SE, 0.5%] to 33.5% [SE, 0.5%]) than those with obesity (from 24.9% [SE, 0.8%] to 30.9% [SE, 1.1%]) (*P* ≤ .001 for interaction).

**Conclusions and Relevance:**

In this cross-sectional study of Korean youths, the estimated proportion of energy intake from ultraprocessed foods increased from 2007 to 2024, indicating that ultraprocessed foods became a more dominant part of Korean youths’ diets, especially among older youths and those without obesity. These findings suggest the need for monitoring and preventive strategies to reduce intake.

## Key Points

**Question:**

What percentage of total energy intake among Korean youths came from ultraprocessed foods between 2007 and 2024?

**Findings:**

In this cross-sectional study of 24 518 Korean youths aged 1 to 18 years, the estimated percentage of energy intake from ultraprocessed foods increased from 25% to 33%, while that from unprocessed or minimally processed foods decreased from 65% to 52%.

**Meaning:**

These findings suggest the need for monitoring and preventive strategies to reduce intake of ultraprocessed foods among Korean youths.

## Teaser

This cross-sectional study characterizes temporal trends in the energy intake contribution of ultraprocessed foods and their subcategories among Korean children and adolescents and examines whether these trends vary by obesity status or demographic subgroup.

## Introduction

Ultraprocessed foods have become a major part of modern diets worldwide, particularly among young populations.^1,2,3,4^ According to Nova, the most frequently used classification system, ultraprocessed foods are often high in sugar, sodium, unhealthy fat, and additives, while low in fiber and essential nutrients, and energy dense; this can lead to various health problems, including obesity and chronic diseases.^5,6^ Sugar-sweetened beverages, packaged snacks, instant noodles, ready-to-eat meals, and processed meats are common examples of ultraprocessed foods.^5^ Growing evidence now links consumption of ultra-processed foods to multiple adverse health outcomes, highlighting the relevance of the Nova framework in nutrition and public health research.^6,7,8^ The public health impact has considerably expanded, and in response, the World Health Organization has recently launched an international expert group to develop guidelines on ultraprocessed foods.^9^

Childhood and adolescent obesity more than tripled worldwide between 1999 and 2021.^10^ The increase was particularly pronounced in East Asia, indicating that Asian regions, where childhood obesity rates were historically lower, experienced some of the steepest growth. In Korea, childhood and adolescent obesity nearly doubled from 10.0% in 2014 to 19.3% in 2021, peaking at 25.9% among boys before declining to 13.8% in 2023, close to pre–COVID-19 levels, with a slight rebound among girls (12.7%).^11^ Abdominal obesity also rose substantially from 7.7% in 2012 to 17.3% in 2021, with a more pronounced increase in boys (7.1% to 22.3%) than in girls (8.4% to 12.1%).^12^

However, previous research has mostly focused on adult populations, and evidence on youths remains relatively underexplored. A multicountry study^13^ reported a positive association between ultraprocessed food consumption and obesity, potentially mediated by higher energy density, free sugar intake, and lower fiber intake, and a study among Canadian children^14^ reported a link between ultraprocessed food intake and adverse adiposity measures. Other studies have focused on total energy intake^4,13,15^ or selected food types, including savory snacks, sweets, fast foods, and sugar-sweetened beverages.^4,16,17^ However, only a few have examined the extent to which children consume ultraprocessed foods and how these patterns have changed over time.^4,15,18^ This study characterized temporal trends in the energy intake contribution of ultraprocessed foods and their subcategories among Korean children and adolescents (hereinafter referred to as youths) aged 1 to 18 years and examined whether these trends varied by obesity status or demographic subgroups from 2007 to 2024 using data from 6 consecutive cycles of the Korea National Health and Nutrition Examination Survey (KNHANES).

## Methods

### Study Population

The KNHANES is a nationally representative, cross-sectional survey conducted by the Korea Disease Control and Prevention Agency (KDCA) to assess the health and nutritional status of the noninstitutionalized Korean population. The KDCA obtained written informed consent from all participants before their participation in KNHANES. The present study was deemed exempt by the Institutional Review Board of the Korea Institute for Health and Social Affairs because only publicly available, deidentified data were used. The study followed the Strengthening the Reporting of Observational Studies in Epidemiology (STROBE) reporting guideline for cross-sectional studies.

The KHNANES uses a complex, multistage probability sampling design.^19^ Data were collected through structured health interviews, health examinations with biomarker measurements, and nutritional surveys. Written informed consent was obtained from all youths aged 1 to 18 years who were able to provide their own responses in the presence of a parent or guardian. Otherwise, the parent or guardian served as a proxy. Detailed descriptions of the KNHANES methodology are available elsewhere.^19^ For this study, we pooled data from the fourth (2007-2009) through the ninth (2022-2024) survey cycles, which were conducted under a continuous, year-round survey system with a standardized operational framework.

Among the 25 291 youths aged 1 to 18 years who completed both the health interviews and nutrition surveys, we excluded those with implausible energy intake based on less than the 1st or greater than the 99th percentile (<500 or >5000 kcal/d [n = 422]) and missing data on covariates (n = 351). The final sample included 24 518 youths (12 797 boys and 11 721 girls).

### Dietary Assessment and Estimation of Ultraprocessed Food Consumption

Dietary data for individuals 1 year and older were collected by trained dieticians using a 1-day 24-hour dietary recall 1 week after the health interviews and examinations. The 24-hour dietary recall collected detailed information on the types, amounts, timing, and locations of all foods and beverages consumed from midnight to midnight, including main meals and snacks. Data were gathered throughout all seasons on weekdays and weekends. Standard household measuring devices and 2-dimensional food models were used to help respondents estimate portion sizes.

We classified foods and beverages using the 5-digit KNHANES food codes mapped to the Nova framework, which evaluates diet based on the degree and purpose of food processing. The Nova framework consists of 4 categories: (1) unprocessed or minimally processed foods, (2) processed culinary ingredients, (3) processed foods, and (4) ultraprocessed foods.^5^ In this study, we applied the classification principles described by Monteiro et al^5^ but adapted them to the Korean context, where mixed dishes are common and multiple ingredients are often combined. The Korean Nova system follows the same rationale but gives greater attention to preserving the food matrix and accounting for traditional eating practices^20,21^ (eTable 1 in Supplement 1). When the distinction between groups 3 and 4 was ambiguous, we considered whether the item existed in the Korean diet before the widespread availability of mass-produced processed foods. We also used KNHANES food codes to separate traditional ingredients from industrial products, and 2 independent researchers (S.J. and J.Y.K.) assigned all items to Nova groups; any disagreements were resolved by checking product information, ingredients, and nutrient profiles, such as sodium or sugar content.^3,22^

### Outcomes

Based on this classification, we estimated the contribution of each Nova group to total energy intake. The primary outcome of interest was the percentage of energy from ultraprocessed foods. For comparison, we also reported the corresponding proportions from other Nova categories. Secondary outcomes included the percentage of energy from ultraprocessed foods across population subgroups and the distribution of energy intake across specific subcategories of ultraprocessed foods (industrial grain foods, ready-to-heat dishes, sweets, sugar-sweetened beverages, flavored dairy products, processed meat, and others).

### Population Subgroups

Trends in the consumption of ultraprocessed foods were further assessed among population subgroups according to age (1-5, 6-12, and 13-18 years), sex (boys and girls), household income level (quartiles 1-2 and quartiles 3-4 of equivalized household income), residential area (urban and rural), and obesity status (no and yes). Age groups were defined in accordance with previous literature^4^ and the school-age classification system in Korea. Obesity was defined as a body mass index for age and sex at or above the 95th percentile based on the 2017 Korean National Growth Chart for Children and Adolescents.^23^

### Statistical Analysis

Weighted proportions with SEs were calculated to describe the sociodemographic characteristics of Korean youths across survey cycles. After adjusting for age, sex, residential area, and household income level, linear regression models were used to estimate the percentage of energy from ultraprocessed foods and other Nova groups across the survey cycle and subcategories of ultraprocessed foods. Linear trends were examined, with the survey cycle modeled as a continuous variable in the survey-weighted linear regression models.

Changes in consumption were calculated as the absolute difference in the percentage of energy between the earliest (2007-2009) and the most recent (2022-2024) KNHANES cycles. To evaluate the extent to which these trends were attributable to demographic changes, main analyses were conducted after adjusting for sociodemographic characteristics (residential area and household income).

To assess potential differences in trends in ultraprocessed food consumption across population subgroups, the Wald *F* tests were used to evaluate interactions between the 2-year survey cycle and each demographic factor. As a sensitivity analysis, we extended the trend analysis to include earlier KNHANES cycles (1998-2005) by incorporating a time variable that reflected the actual intervals between survey cycles. All analyses applied KNHANES survey weights covering the nutrition survey to account for the complex sampling design and incorporated survey design variables for clustering and stratification. All analyses were conducted using SAS, version 9.4 (SAS Institute Inc), with PROC SURVEY procedures; statistical significance was defined as a 2-sided α = .05.

## Results

### Population Characteristics

Among the 24 518 youths included, the weighted mean (SD) age was 10.2 (5.1) years; 12 797 (52.2%) were boys and 11 721 (47.8%) were girls. From the 2007-2009 to 2022-2024 cycles, the weighted proportion (SE) of youths living in urban areas increased from 82.7% (2.3%) to 85.6% (2.3%), whereas the weighted proportion (SE) of youths living in rural areas decreased from 17.3% (2.3%) to 14.4% (2.3%) (Table 1). The weighted proportion (SE) of youths with a lower household income (quartiles 1-2) decreased from 35.5% (1.4%) to 32.8% (1.4%).

**Table 1.** Demographic Characteristics of Korean Youths Aged 1 to 18 Years by KNHANES Cycle, 2007 to 2024

Characteristic	KNHANES cycle, weighted % (SE)^a^					
	2007-2009 (n = 5424)	2010-2012 (n = 5014)	2013-2015 (n = 4194)	2016-2018 (n = 4053)	2019-2021 (n = 3048)	2022-2024 (n = 2785)
Age group, y						
1-5	20.7 (0.8)	21.6 (0.9)	24.1 (0.9)	24.7 (0.9)	23.5 (1.0)	19.4 (0.9)
6-12	41.8 (0.9)	38.4 (0.9)	37.4 (0.9)	37.6 (0.9)	41.7 (1.1)	43.2 (1.0)
13-18	37.5 (1.1)	40.0 (1.1)	38.4 (1.1)	37.6 (1.1)	34.8 (1.3)	37.4 (1.3)
Sex						
Boys	53.2 (0.9)	53.3 (0.9)	52.2 (0.9)	51.6 (0.9)	52.1 (1.0)	51.7 (1.0)
Girls	46.8 (0.9)	46.7 (0.9)	47.8 (0.9)	48.4 (0.9)	47.9 (1.0)	48.3 (1.0)
Residential area						
Urban	82.7 (2.3)	83.9 (1.9)	83.5 (2.0)	87.6 (1.8)	88.5 (1.6)	85.6 (2.3)
Rural	17.3 (2.3)	16.1 (1.9)	16.5 (2.0)	12.4 (1.8)	11.5 (1.6)	14.4 (2.3)
Household income^b^						
Q1	10.4 (0.8)	12.6 (1.0)	9.9 (0.7)	8.9 (0.7)	6.6 (0.8)	6.2 (0.7)
Q2	25.2 (1.1)	31.5 (1.3)	27.7 (1.2)	27.8 (1.2)	28.6 (1.4)	26.7 (1.3)
Q3	34.2 (1.1)	31.4 (1.1)	34.7 (1.2)	33.4 (1.2)	35.2 (1.5)	37.4 (1.5)
Q4	30.2 (1.5)	24.5 (1.2)	27.7 (1.3)	29.9 (1.4)	29.6 (1.6)	29.7 (1.6)
Obesity^c^						
No	86.4 (0.6)	84.8 (0.6)	85.8 (0.6)	84.8 (0.7)	81.5 (1.0)	82.3 (0.9)
Yes	13.6 (0.6)	15.2 (0.6)	14.2 (0.6)	15.2 (0.7)	18.5 (1.0)	17.7 (0.9)

Abbreviations: KNHANES, Korea National Health and Nutrition Examination Survey; Q, quartile.

^a^ Sample sizes are unweighted and all other estimates are weighted.

^b^ Quartiles 1 and 2 indicate lower household income; quartiles 3 and 4, higher household income.

^c^ Defined as body mass index for age and sex at or above 95th percentile based on the 2017 Korean National Growth Chart for Children and Adolescents.

### Trends in Ultraprocessed Food Consumption and Other Nova Group Consumption

From 2007 to 2024, there was no significant linear trend in daily total energy intake (1616.0 kcal [SE, 13.3 kcal] to 1668.0 kcal [SE, 14.0 kcal]; *P* = .94 for trend). During the same period, the percentage of energy from minimally processed foods decreased from 64.8% (SE, 0.5%) to 51.8% (SE, 0.4%) (difference, 13.0 percentage points [pp] [SE, 0.6 pp]; *P* < .001 for trend), whereas that from ultraprocessed foods increased from 24.6% (SE, 0.5%) to 33.0% (SE, 0.5%) (difference, 8.4 pp [SE, 0.7 pp]; *P* < .001 for trend). The percentage of energy from processed culinary ingredients increased from 3.8% (SE, 0.1%) to 4.6% (SE, 0.1%), while that from processed foods increased from 6.7% (SE, 0.2%) to 10.5% (SE, 0.3%) (*P* < .001 for trend for both) (Table 2).

**Table 2.** Trends in Percentage of Energy Intake From Nova Food Groups Among Korean Youths Aged 1 to 18 Years by KNHANES Cycle, 2007 to 2024

Energy intake source	KNHANES cycle, weighted mean (SE)^a^						*P* value for linear trend^b^	2022-2024 vs 2007-2009, mean difference (SE), pp
	2007-2009 (n = 5424)	2010-2012 (n = 5014)	2013-2015 (n = 4194)	2016-2018 (n = 4053)	2019-2021 (n = 3048)	2022-2024 (n = 2785)		
Total kcal	1616.0 (13.3)	1803.4 (14.4)	1810.6 (13.4)	1767.7 (14.1)	1684.2 (15.6)	1668.0 (14.0)	.94	52.1 (19.4)
Nova groups, %								
Unprocessed or minimally processed foods	64.8 (0.5)	61.2 (0.4)	56.4 (0.4)	54.0 (0.4)	53.1 (0.5)	51.8 (0.4)	<.001	−13.0 (0.6)
Processed culinary ingredients	3.8 (0.1)	3.8 (0.1)	4.1 (0.1)	3.9 (0.1)	4.2 (0.1)	4.6 (0.1)	<.001	0.8 (0.1)
Processed foods	6.7 (0.2)	6.8 (0.2)	8.3 (0.2)	9.6 (0.2)	10.2 (0.3)	10.5 (0.3)	<.001	3.8 (0.3)
Ultraprocessed foods	24.6 (0.5)	28.3 (0.4)	31.2 (0.4)	32.3 (0.4)	32.4 (0.5)	33.0 (0.5)	<.001	8.4 (0.7)

Abbreviations: KNHANES, Korea National Health and Nutrition Examination Survey; pp, percentage points.

^a^ Data were obtained using a linear regression model after adjusting for age, sex, residential area, and household income levels and weighted.

^b^ Assessed by modeling the survey cycle as a continuous variable in survey-weighted linear regression models.

### Trends in Consumption of Ultraprocessed Food Subgroups

**Figure 1.**
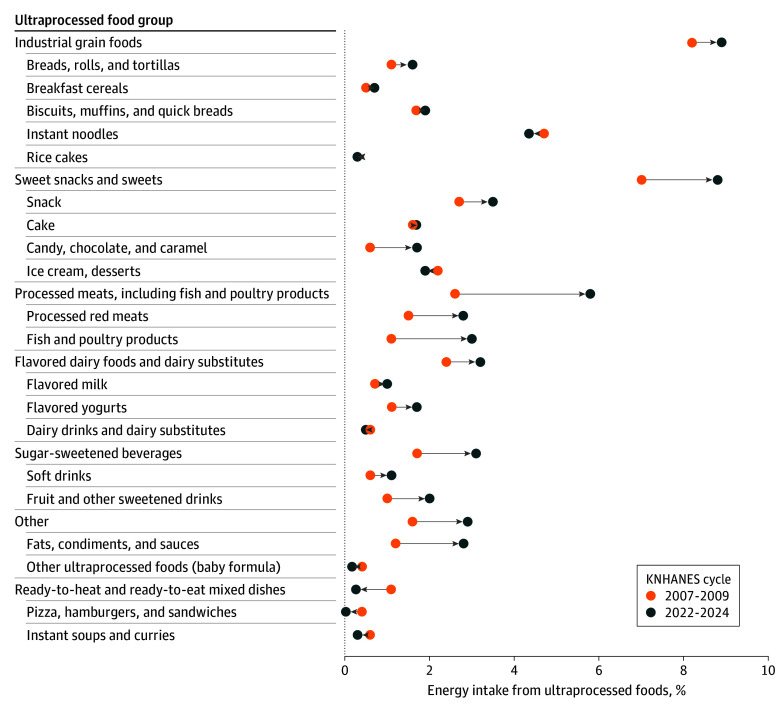
Dot Plot of Change in Percentage of Total Energy Intake Contribution of Ultraprocessed Foods Between the 2007-2009 and 2022-2024 Korea National Health and Nutrition Examination Survey (KNHANES) Cycles Estimated means and SEs were obtained using a weighted linear regression model after adjusting for age, sex, residential area, and monthly household income. Rightward-pointing arrows indicate increased consumption over time; leftward-pointing arrows indicate decreased consumption.

In the 2022-2024 cycle, the subgroups of ultraprocessed foods that contributed the largest percentage of energy were industrial grain foods (8.9% [SE, 0.3%]), followed by sweet snacks and sweets (8.8% [SE, 0.3%]), processed meats that included fish and poultry products (5.8% [SE, 0.2%]), and flavored dairy foods and substitutes (3.2% [SE, 0.1%]) (Table 3). From the 2007-2009 to 2022-2024 cycles, the percentage of energy from consumption of sweet snacks and sweets increased from 7.0% (SE, 0.2%) to 8.8% (SE, 0.3%), that from the processed meats subgroup increased from 2.6% (SE, 0.1%) to 5.8% (SE, 0.2%), that from flavored dairy products increased from 2.4% (SE, 0.1%) to 3.2% (SE, 0.1%), that from sugar-sweetened beverages increased from 1.7% (SE, 0.1%) to 3.1% (SE, 0.1%), and that from the other subgroup increased from 1.6% (SE, 0.1%) to 2.9% (SE, 0.1%) (*P* < .001 for trend for all). The percentage of energy from ready-to-heat and ready-to-eat mixed dishes decreased from 1.1% (SE, 0.1%) to 0.3% (SE, 0.04%) (*P* = .001 for trend); the increase in in industrial grain food consumption from 8.2% (SE, 0.3%) to 8.9% (SE, 0.3%) (*P* = .14 for trend) was not significant (Table 3 and Figure 1).

**Table 3.** Trends in Percentage of Energy Intake From Ultraprocessed Foods by Subcategories Among Korean Youths Aged 1 to 18 Years by KNHANES Cycle, 2007 to 2024

Ultraprocessed food subcategory	KNHANES cycle, weighted mean (SE), % kcal^a^						*P* value for linear trend^b^	2022-2024 vs 2007-2009, difference, mean (SE), pp
	2007-2009 (n = 5424)	2010-2012 (n = 5014)	2013-2015 (n = 4194)	2016-2018 (n = 4053)	2019-2021 (n = 3048)	2022-2024 (n = 2785)		
Total	24.6 (0.5)	28.3 (0.4)	31.2 (0.4)	32.3 (0.4)	32.4 (0.5)	33.0 (0.5)	<.001	8.4 (0.7)
Industrial grain foods	8.2 (0.3)	8.5 (0.3)	8.8 (0.3)	9.0 (0.2)	8.4 (0.3)	8.9 (0.3)	.14	0.7 (0.4)
Breads, rolls, and tortillas	1.1 (0.1)	1.2 (0.1)	1.3 (0.1)	1.1 (0.1)	1.2 (0.1)	1.6 (0.1)	.002	0.5 (0.1)
Breakfast cereals	0.5 (0.04)	0.6 (0.05)	0.5 (0.04)	0.6 (0.05)	0.7 (0.06)	0.7 (0.06)	<.001	0.3 (0.1)
Muffins and quick breads	1.7 (0.1)	2.2 (0.1)	2.3 (0.1)	2.1 (0.1)	1.8 (0.1)	1.9 (0.1)	.96	0.2 (0.2)
Instant noodles	4.7 (0.2)	4.2 (0.2)	4.5 (0.2)	4.9 (0.2)	4.5 (0.2)	4.4 (0.2)	.90	−0.3 (0.3)
Rice cakes	0.3 (0.05)	0.3 (0.05)	0.2 (0.04)	0.2 (0.03)	0.2 (0.03)	0.3 (0.04)	.09	−0.03 (0.1)
Sweet snacks and sweets	7.0 (0.2)	8.4 (0.2)	9.1 (0.2)	9.0 (0.2)	8.6 (0.2)	8.8 (0.3)	<.001	1.8 (0.3)
Snack	2.7 (0.1)	3.4 (0.1)	3.5 (0.1)	3.5 (0.1)	3.3 (0.1)	3.5 (0.2)	.003	0.7 (0.2)
Cake	1.6 (0.1)	1.5 (0.1)	1.6 (0.1)	1.6 (0.1)	1.2 (0.1)	1.7 (0.1)	.91	0.1 (0.2)
Candy, chocolate, caramel	0.6 (0.1)	1.2 (0.1)	1.5 (0.1)	1.6 (0.1)	1.6 (0.1)	1.7 (0.1)	<.001	1.2 (0.1)
Ice cream, desserts	2.2 (0.1)	2.4 (0.1)	2.6 (0.1)	2.3 (0.1)	2.5 (0.1)	1.9 (0.1)	.41	−0.3 (0.2)
Processed meats	2.6 (0.1)	2.9 (0.1)	3.7 (0.1)	4.5 (0.2)	5.6 (0.2)	5.8 (0.2)	<.001	3.2 (0.2)
Red meats	1.5 (0.1)	1.5 (0.1)	1.8 (0.1)	1.9 (0.1)	2.8 (0.1)	2.8 (0.1)	<.001	1.3 (0.2)
Fish and poultry products	1.1 (0.1)	1.4 (0.1)	2.0 (0.1)	2.5 (0.1)	2.8 (0.2)	3.0 (0.2)	<.001	1.9 (0.2)
Flavored dairy foods and dairy substitutes	2.4 (0.1)	3.0 (0.1)	2.9 (0.1)	3.0 (0.1)	2.8 (0.1)	3.2 (0.1)	<.001	0.8 (0.2)
Flavored milk	0.7 (0.1)	0.7 (0.1)	0.7 (0.1)	0.9 (0.1)	0.8 (0.1)	1.0 (0.1)	.001	0.3 (0.1)
Flavored yogurts	1.1 (0.1)	1.5 (0.1)	1.5 (0.1)	1.7 (0.1)	1.5 (0.1)	1.7 (0.1)	<.001	0.6 (0.1)
Dairy drinks and dairy substitutes	0.6 (0.1)	0.8 (0.1)	0.7 (0.1)	0.5 (0.04)	0.5 (0.1)	0.5 (0.04)	.001	−0.1 (0.1)
Sugar-sweetened beverages	1.7 (0.1)	2.7 (0.1)	3.3 (0.1)	3.1 (0.1)	3.0 (0.1)	3.1 (0.1)	<.001	1.4 (0.1)
Soft drinks	0.6 (0.1)	1.0 (0.1)	1.2 (0.1)	1.3 (0.1)	1.3 (0.1)	1.1 (0.1)	<.001	0.4 (0.1)
Fruit and other sweetened drinks	1.0 (0.1)	1.7 (0.1)	2.1 (0.1)	1.8 (0.1)	1.6 (0.1)	2.0 (0.1)	<.001	1.0 (0.1)
Other	1.6 (0.1)	1.7 (0.1)	1.9 (0.1)	2.4 (0.1)	2.7 (0.1)	2.9 (0.1)	<.001	1.3 (0.1)
Fats, condiments, and sauces	1.2 (0.1)	1.4 (0.05)	1.8 (0.1)	2.2 (0.1)	2.6 (0.1)	2.8 (0.1)	<.001	1.5 (0.1)
Other ultraprocessed foods (eg, baby formula)	0.4 (0.05)	0.3 (0.06)	0.1 (0.03)	0.1 (0.02)	0.2 (0.04)	0.2 (0.04)	<.001	−0.2 (0.1)
Ready-to-heat and ready-to-eat mixed dishes	1.1 (0.1)	1.1 (0.1)	1.4 (0.1)	1.3 (0.1)	1.3 (0.1)	0.3 (0.04)	.001	−0.8 (0.1)
Pizza, hamburgers, and sandwiches	0.4 (0.1)	0.5 (0.1)	1.0 (0.1)	1.0 (0.1)	1.0 (0.1)	0.02 (0.03)	.73	−0.4 (0.1)
Instant soups and curries	0.6 (0.1)	0.5 (0.1)	0.4 (0.04)	0.3 (0.02)	0.3 (0.03)	0.3 (0.03)	<.001	−0.3 (0.1)

Abbreviations: KNHANES, Korea National Health and Nutrition Examination Survey; pp, percentage points.

^a^ Data were obtained using a linear regression model after adjusting for age, sex, residential area, and household income levels and weighted.

^b^ Assessed by modeling the survey cycle as a continuous variable in survey-weighted linear regression models.

Among the sweet snacks and sweets subgroup, the percentage of energy increased for snack food (2.7% [SE, 0.1%] to 3.5% [SE, 0.2%]) (*P* = .003 for trend) and for candy, chocolate, and caramel (0.6% [SE, 0.1%] to 1.7% [SE, 0.1%]) (*P* < .001 for trend). Among processed meats, the percentage of energy increased for both red meats (1.5% [SE, 0.1%] to 2.8% [SE, 0.1%]) (*P* < .001 for trend) and fish and poultry products (1.1% [SE, 0.1%] to 3.0% [SE, 0.2%]) (*P* < .001 for trend). Among the flavored dairy foods and dairy substitutes, the percentage of energy increased for flavored milk (0.7% [SE, 0.1%] to 1.0% [SE, 0.1%]) (*P* = .001 for trend) and flavored yogurts (1.1% [SE, 0.1%] to 1.7% [SE, 0.1%]) (*P* < .001 for trend). In the sugar-sweetened beverages subgroups, the percentage of energy increased for both soft drinks (0.6% [SE, 0.1%] to 1.1% [SE, 0.1%]) (*P* < .001 for trend) and fruit and other sweetened drinks (1.0% [SE, 0.1%] to 2.0% [SE, 0.1%]) (*P* < .001 for trend). Among the other subgroups, the percentage of energy from fats, condiments, and sauces increased (1.2% [SE, 0.1%] to 2.8% [SE, 0.1%]) (*P* < .001 for trend). The percentage of energy decreased for dairy drinks and substitutes (0.6% [SE, 0.1%] to 0.5% [SE, 0.04%]), baby formula (0.4% [SE, 0.1%] to 0.2% [SE, 0.04%]), and instant soups and curries (0.6% [SE, 0.1%] to 0.3% [SE, 0.03%]) (*P* < .001 for trend for all) (Table 3).

### Trends in Population Subgroups and Sensitivity Analysis

**Figure 2.**
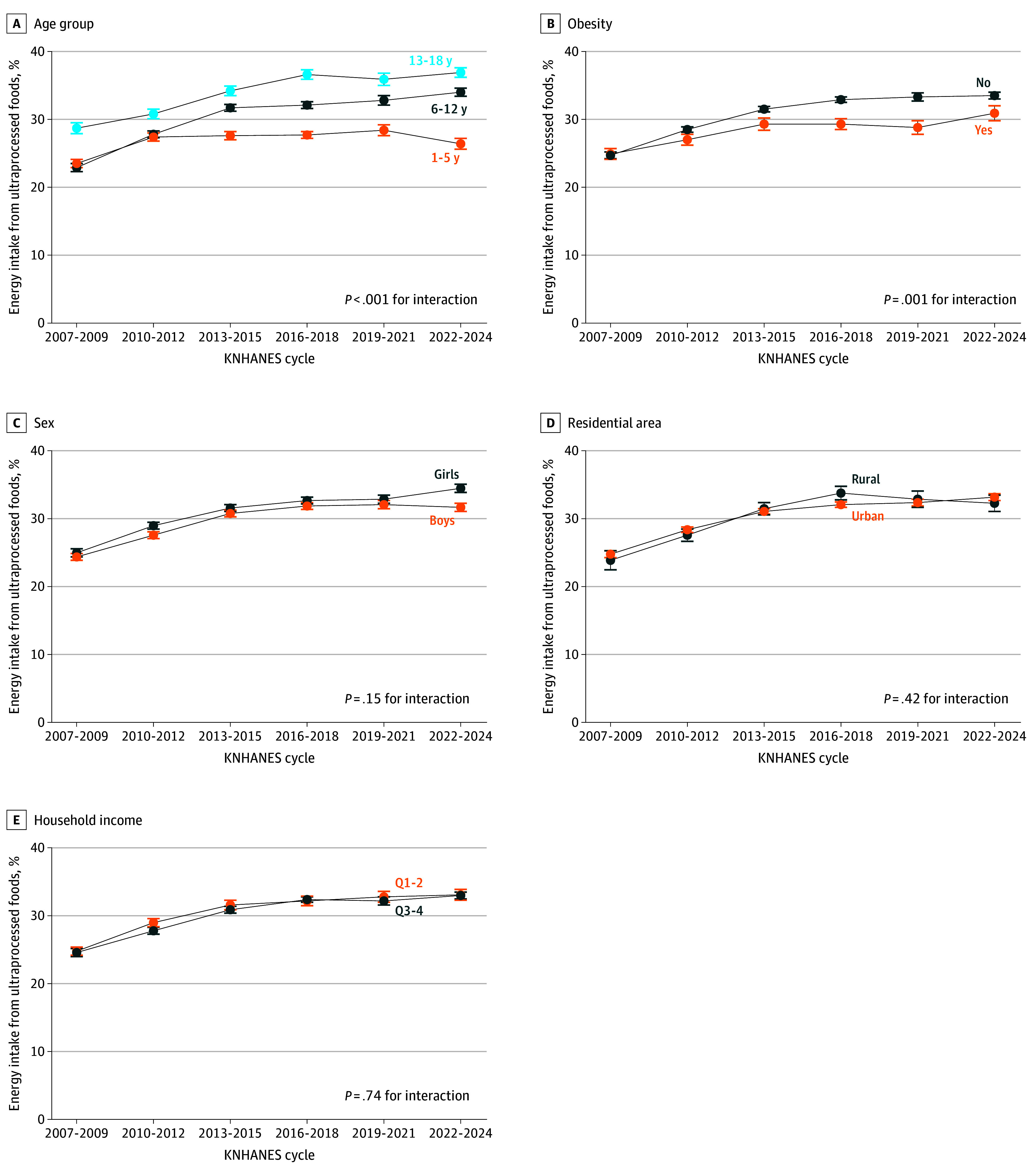
Line Graphs of Trends in Percentage of Energy Intake From Ultraprocessed Foods Among Population Subgroups of Korean Youths Aged 1 to 18 Years by Korea National Health and Nutrition Examination Survey (KNHANES) Cycle Estimated means were obtained using a weighted linear regression model after adjusting for age, sex, residential area, and monthly household income except for the corresponding subgroup variables. Household income quartiles (Q) are stratified by lower (Q1-2) and higher (Q3-4) income. Error bars indicate SEs.

From the 2007-2009 to 2022-2024 cycles, the percentage of energy from ultraprocessed foods increased across all population subgroups; however, the increase was higher among those aged 6 to 12 years (from 22.9% [SE, 0.6%] to 34.0% [SE, 0.6%]; difference, 11.2 pp [SE, 0.8 pp]) and those aged 13 to 18 years (from 28.7% [SE, 0.8%] to 36.9% [SE, 0.7%]; difference, 8.2 pp [SE, 1.1 pp]) than the increase among those aged 1 to 5 years (from 23.5% [SE, 0.6%] to 26.4% [SE, 0.9%]; difference, 2.8 pp [SE, 1.0 pp]) (*P* < .001 for interaction). The increase was higher among youths without obesity (from 24.7% [SE, 0.5%] to 33.5% [SE, 0.5%]; difference, 8.9 pp [SE, 0.7 pp]) than among those with obesity (from 24.9% [SE, 0.8%] to 30.9% [SE, 1.1%]; difference, 6.0 pp [SE, 1.4 pp]) (*P* = .001 for interaction) (Figure 2). In the 2022-2024 cycle, older youths and those without obesity consumed a higher percentage of energy from ultraprocessed foods than their counterparts. Results from sensitivity analyses including earlier survey cycles (1998-2005) were consistent with the main findings (eTables 2 and 3 and eFigures 1 and 2 in Supplement 1).

## Discussion

In this analysis of nationally representative data, Korean youths obtained 33.0% of their daily energy from ultraprocessed foods from 2022 to 2024. The percentage of energy from ultraprocessed foods increased by 8.4 pp from 2007 to 2024, whereas that from minimally processed foods decreased by 13.0 pp during the same period. When looking at the subcategories, a pronounced increase in processed meat, sweet snacks and sweets, sugar-sweetened beverages, and flavored dairy foods and substitutes and a decrease in ready-to-heat and ready-to-eat mixed dishes were observed. Older youths and those without obesity showed a greater increase in the percentage of energy from consumption of ultraprocessed foods than their counterparts.

The percentage of energy from ultraprocessed foods among Korean youths was lower than that in the US and the United Kingdom (UK), but higher than that in Taiwan. In 2011, youths in the US and UK obtained 67.0%^4^ and 65.9%^18^ of their energy, respectively, from ultraprocessed foods, while Taiwanese adolescents consumed 25.0%.^15^ Although Korean youths seem to consume relatively lower levels of ultraprocessed foods, the increasing consumption trend is concerning. The percentage of energy from ultraprocessed foods decreased by 4.8% among UK adolescents from 2008 to 2019^18^ and increased by 5.6% among US youths from 1999 to 2018^4^ and by 4.0% among Taiwanese adolescents from 1993 to 2011.^15^ In our study, ultraprocessed food consumption among Korean youths increased by 8.4 pp, indicating that even at a relatively lower consumption level, the increasing trend is alarming and should be interpreted with caution.

The pronounced increases in several subcategories of ultraprocessed foods (eg, processed meats; fats, condiments, and sauces; and sugar-sweetened beverages) may reflect broader changes in the food environment, including greater availability and commercialization of prepared and highly seasoned foods associated with the expansion of food delivery services^24^ and the high accessibility of convenience stores in Korea, which may increase exposure to sugar-sweetened beverages.^25,26^ At the same time, these trends may also be influenced by market expansion, including greater product variety, as well as potential changes in food classification over time, which should be considered when interpreting temporal changes in these food subcategories.

This study observed a greater increase in the consumption of ultraprocessed foods among school-aged children and adolescents than preschool-aged children. Adolescents may be more vulnerable to higher ultraprocessed food consumption because this developmental stage is characterized by increased independence from parental control over food choices, greater exposure to food marketing, and more frequent eating outside of parental supervision.^27,28^ In particular, greater exposure to food-related media such as mukbangs (online videos in which hosts consume food) has been associated with unhealthy eating behaviors, including skipping meals, late-night snacking, higher intake of sweets, Korean-style street food, sweetened beverages, and out-of-home meals in Korean children and adolescents aged 9 to 18 years.^29^ This speculation needs to be further studied.

When stratified by obesity status, the increase in ultraprocessed food intake was more pronounced among youths without obesity, and they consistently had slightly higher levels than those with obesity, except during the 2007-2009 cycle. Although the types of ultraprocessed foods, such as conventional or premium (beneficial to health), was not identified in our study, youths without obesity might consume more premium or healthy ultraprocessed foods, such as yogurt without too much sugar or alternative meat-based sausages, while youths with obesity consume mostly conventional ultraprocessed foods. This possibility remains speculative and warrants further investigation.

Alternatively, genetic and phenotypic differences may influence how individuals metabolize and respond to energy-dense foods, leading some children to gain weight more easily even when consuming similar diets.^30^ Parental influence may also contribute, as parents of children with obesity could be more attentive to their children’s diet and actively restrict the intake of highly processed foods.^31^ Future studies integrating genetic profiles and longitudinal data are needed to clarify these mechanisms.

To the best of our knowledge, this is the first study to characterize temporal trends in energy intake from ultraprocessed foods and their subgroups among children and adolescents in Korea. This study also provides the most recent analysis of the trends in ultraprocessed food consumption in Asian countries. Additionally, the use of nationally representative dietary data collected through standardized protocols can provide generalizability of the findings to Korean youths.

### Limitations

This study had some limitations. First, youths’ responses to the 24-hour dietary recall may be subject to measurement errors and underreporting issues, and the 24-hour dietary recall may not perfectly assess individuals’ usual intake due to day-to-day variations. In addition, the distribution of weekday vs weekend recalls may have varied across survey cycles, and a single-day recall may be insufficient to capture episodically consumed ultraprocessed foods. Second, variation in sample size across survey cycles may have affected the precision of the estimates; however, the application of survey weights and pooling of multiple cycles help to mitigate potential bias. Third, our study participants were Korean youths, limiting the generalizability of our findings to settings with other races and ethnicities, adults, or other countries. Fourth, the Nova classification system may have weaknesses, such as its simplistic definition that overlooks the food’s characteristics.^32^ Specifically, not all ultraprocessed foods, such as plain yogurt, whole-grain bread, and granola without sugar, are inherently harmful to health^33,34,35^; however, they are classified as ultraprocessed foods in the Nova system. Although Nova is the most widely used classification method,^7^ it may not accurately distinguish between these characteristics. In addition, the databases used in the Korean studies may not have fully captured the rapidly expanding variety of ultraprocessed foods, potentially compromising the accuracy of the estimates.^36^ In particular, the sharp decline in consumption of pizza, hamburgers, and sandwiches in the most recent survey cycles may reflect incomplete capture of newly introduced products rather than true reductions in consumption. Thus, cautious interpretation is warranted.

## Conclusions

In this cross-sectional study of Korean youths, the estimated proportion of energy intake from ultraprocessed foods increased from 2007 to 2024, indicating that ultraprocessed foods became a more dominant part of Korean youth diet, especially among older youths and those without obesity. These findings highlight a major shift in dietary patterns among Korean youths toward ultraprocessed foods, which may have long-term health consequences. Even among youths without obesity, high intake of ultraprocessed foods indicates a need for monitoring and preventive strategies, and not just for those individuals who are already at risk. Policy measures, such as nutrition education and regulation of ultraprocessed food marketing to youths, should be considered to curb this trend.

## Notes

Supplement 1. **eTable 1.** Korean NOVA Classification System Based on Product Characteristics **eTable 2.** Trends in Multivariable-Adjusted Percentage of Energy Intake From Nova Food Groups Among Korean Youths Aged 1 to 18 Years by KNHANES Cycle, 1998 to 2024 **eTable 3.** Trends in Multivariable-Adjusted Percentage of Energy Intake From Ultraprocessed Foods by Subcategories Among Korean Youths Aged 1 to 18 Years by KNHANES Cycle, 1998 to 2024 **eFigure 1.** Multivariable-Adjusted Changes in Percentage of Energy Intake From Subgroups of Ultraprocessed Food Consumption Among Korean Youths Aged 1 to 18 Years by KNHANES Cycle, 1998 to 2024 **eFigure 2.** Trends in Multivariable-Adjusted Percentage of Energy Intake From Ultraprocessed Foods Among Population Subgroups of Korean Youths Aged 1 to 18 Years by KNHANES Cycle, 1998 to 2024

Supplement 2.
Data Sharing Statement

## References

[1] Baker P, Machado P, Santos T, . Ultra-processed foods and the nutrition transition: global, regional and national trends, food systems transformations and political economy drivers. Obes Rev. 2020;21(12):e13126. doi:10.1111/obr.13126 32761763

[2] Wood B, Garton K, Milsom P, . Using a systems thinking approach to map the global rise of ultra-processed foods in population diets. Obes Rev. 2025;26(4):e13877. doi:10.1111/obr.13877 39627009 PMC11884965

[3] Jung S, Kim JY, Park S. Eating patterns in Korean adults, 1998-2018: increased energy contribution of ultra-processed foods in main meals and snacks. Eur J Nutr. 2024;63(1):279-289. doi:10.1007/s00394-023-03258-x 37999737 PMC10799128

[4] Wang L, Martínez Steele E, Du M, . Trends in consumption of ultraprocessed foods among US youths aged 2-19 years, 1999-2018. JAMA. 2021;326(6):519-530. doi:10.1001/jama.2021.10238 34374722 PMC8356071

[5] Monteiro CA, Cannon G, Levy RB, . Ultra-processed foods: what they are and how to identify them. Public Health Nutr. 2019;22(5):936-941. doi:10.1017/S1368980018003762 30744710 PMC10260459

[6] Lane MM, Gamage E, Du S, . Ultra-processed food exposure and adverse health outcomes: umbrella review of epidemiological meta-analyses. BMJ. 2024;384:e077310. doi:10.1136/bmj-2023-077310 38418082 PMC10899807

[7] Moubarac JC, Parra DC, Cannon G, Monteiro CA. Food classification systems based on food processing: significance and implications for policies and actions: a systematic literature review and assessment. Curr Obes Rep. 2014;3(2):256-272. doi:10.1007/s13679-014-0092-0 26626606

[8] Monteiro CA, Astrup A. Does the concept of “ultra-processed foods” help inform dietary guidelines, beyond conventional classification systems? YES. Am J Clin Nutr. 2022;116(6):1476-1481. doi:10.1093/ajcn/nqac122 35670127

[9] Guideline Development Group for ultra-processed foods World Health Organization. 2025. Accessed December 3, 2025. https://www.who.int/groups/guideline-development-group-for-ultra-processed-foods

[10] GBD 2021 Adolescent BMI Collaborators. Global, regional, and national prevalence of child and adolescent overweight and obesity, 1990-2021, with forecasts to 2050: a forecasting study for the Global Burden of Disease Study 2021. Lancet. 2025;405(10481):785-812. doi:10.1016/S0140-6736(25)00397-6 40049185 PMC11920006

[11] 2025 Obesity fact sheet. Korean Society for the Study of Obesity. 2025. Accessed October 11, 2025. https://general.kosso.or.kr/html/user/core/view/reaction/main/kosso/inc/data/2025_Obesity_Fact_sheet_web_kor.pdf

[12] Jeong SM, Jung JH, Yang YS, ; Taskforce Team of the Obesity Fact Sheet of the Korean Society for the Study of Obesity. 2023 Obesity Fact Sheet: prevalence of obesity and abdominal obesity in adults, adolescents, and children in Korea from 2012 to 2021. J Obes Metab Syndr. 2024;33(1):27-35. doi:10.7570/jomes24012 38531533 PMC11000515

[13] Neri D, Steele EM, Khandpur N, ; NOVA Multi-Country Study Group on Ultra-Processed Foods, Diet Quality and Human Health. Ultraprocessed food consumption and dietary nutrient profiles associated with obesity: a multicountry study of children and adolescents. Obes Rev. 2022;23(suppl 1):e13387. doi:10.1111/obr.13387 34889015

[14] Chen ZH, Mousavi S, Mandhane PJ, . Ultraprocessed food consumption and obesity development in Canadian children. JAMA Netw Open. 2025;8(1):e2457341. doi:10.1001/jamanetworkopen.2024.57341 39888617 PMC11786234

[15] Chen YC, Huang YC, Lo YC, Wu HJ, Wahlqvist ML, Lee MS. Secular trend towards ultra-processed food consumption and expenditure compromises dietary quality among Taiwanese adolescents. Food Nutr Res. 2018;62. doi:10.29219/fnr.v62.1565 30258346 PMC6150927

[16] Petridi E, Karatzi K, Magriplis E, Charidemou E, Philippou E, Zampelas A. The impact of ultra-processed foods on obesity and cardiometabolic comorbidities in children and adolescents: a systematic review. Nutr Rev. 2024;82(7):913-928. doi:10.1093/nutrit/nuad095 37550263

[17] Al Hourani H, Shhadeh HA, Al-Jawaldeh A. Association between consumption of ultra processed foods and obesity among Jordanian children and adolescents. Sci Rep. 2025;15(1):9326. doi:10.1038/s41598-025-93506-3 40102562 PMC11920511

[18] Chavez-Ugalde IY, de Vocht F, Jago R, . Ultra-processed food consumption in UK adolescents: distribution, trends, and sociodemographic correlates using the National Diet and Nutrition Survey 2008/09 to 2018/19. Eur J Nutr. 2024;63(7):2709-2723. doi:10.1007/s00394-024-03458-z 39014218 PMC11490440

[19] Kweon S, Kim Y, Jang MJ, . Data resource profile: the Korea National Health and Nutrition Examination Survey (KNHANES). Int J Epidemiol. 2014;43(1):69-77. doi:10.1093/ije/dyt228 24585853 PMC3937975

[20] Park HJ, Park S, Kim JY. Development of Korean NOVA food classification and estimation of ultra-processed food intake among adults: using 2018 Korea National Health and Nutrition Examination Survey. Korean J Community Nutr. 2022;27(6):455-467. doi:10.5720/kjcn.2022.27.6.455

[21] UPF Working Group. Nutritional epidemiology lab. 2025. Accessed November 19, 2025. https://www.nutritional-epidemiology.org/upf

[22] Jung S, Park S, Kim JY. Comparison of dietary share of ultra-processed foods assessed with a FFQ against a 24-h dietary recall in adults: results from KNHANES 2016. Public Health Nutr. 2022;25(5):1-10. doi:10.1017/S1368980022000179 35042567 PMC9991629

[23] Kim JH, Yun S, Hwang SS, ; Committee for the Development of Growth Standards for Korean Children and Adolescents; Committee for School Health and Public Health Statistics, the Korean Pediatric Society; Division of Health and Nutrition Survey, Korea Centers for Disease Control and Prevention. The 2017 Korean National Growth Charts for Children and Adolescents: development, improvement, and prospects. Korean J Pediatr. 2018;61(5):135-149. doi:10.3345/kjp.2018.61.5.135 29853938 PMC5976563

[24] Lee SH, Kim SR. The role of Korean food delivery apps as a third-party logistics provider: focusing on self-determination theory. Global Business Finance Rev. 2024;29(5):187-201. doi:10.17549/gbfr.2024.29.5.187

[25] Yoon NH, Shon C. Convenience store use and the health of urban adolescents in Seoul, South Korea. Int J Environ Res Public Health. 2020;17(18):6486. doi:10.3390/ijerph17186486 32899954 PMC7558625

[26] Laska MN, Hearst MO, Forsyth A, Pasch KE, Lytle L. Neighbourhood food environments: are they associated with adolescent dietary intake, food purchases and weight status? Public Health Nutr. 2010;13(11):1757-1763. doi:10.1017/S1368980010001564 20529405 PMC3119051

[27] Brody R, Colombet Z, van Sluijs E, Chavez-Ugalde Y. Examining the influence of socio-economic factors on ultra-processed food consumption patterns of UK adolescents. Public Health Nutr. 2025;28(1):e140. doi:10.1017/S136898002510075X 40708201 PMC12516625

[28] Rauber F, Martins CA, Azeredo CM, Leffa PS, Louzada MLC, Levy RB. Eating context and ultraprocessed food consumption among UK adolescents. Br J Nutr. 2022;127(1):112-122. doi:10.1017/S0007114521000854 33691816

[29] Jang E, Ko E, Sim J, Jeong M, Park S. *Mukbang* media: correlations with the dietary behavior of children and adolescents in Korea. Nutr Res Pract. 2024;18(5):674-686. doi:10.4162/nrp.2024.18.5.674 39398882 PMC11464279

[30] Viljakainen H, Sorlí JV, Dahlström E, Agrawal N, Portolés O, Corella D. Interaction between genetic susceptibility to obesity and food intake on BMI in Finnish school-aged children. Sci Rep. 2023;13(1):15265. doi:10.1038/s41598-023-42430-5 37709841 PMC10502078

[31] Prates CB, Passos MAZ, Masquio DCL. Parental feeding practices and ultra-processed food consumption in preschool children. Rev Nutr. 2022;35:e210269. doi:10.1590/1678-9865202235e210269

[32] Gibney MJ, Forde CG, Mullally D, Gibney ER. Ultra-processed foods in human health: a critical appraisal. Am J Clin Nutr. 2017;106(3):717-724. doi:10.3945/ajcn.117.160440 28793996

[33] Monteiro CA, Rezende LFM. Are all ultra-processed foods bad for health? Lancet Reg Health Eur. 2024;46:101106. doi:10.1016/j.lanepe.2024.101106 39493247 PMC11530752

[34] Louie JCY. Are all ultra-processed foods bad? a critical review of the NOVA classification system. Proc Nutr Soc. Published online August 4, 2025. doi:10.1017/S0029665125100645 40757421

[35] Dicken SJ, Jassil FC, Brown A, . Ultraprocessed or minimally processed diets following healthy dietary guidelines on weight and cardiometabolic health: a randomized, crossover trial. Nat Med. 2025;31(10):3297-3308. doi:10.1038/s41591-025-03842-0 40760353 PMC12532614

[36] Jung S, Kim JY, Park S, Lee JE; UPF Working Group. Potential misclassification of ultra-processed foods across studies and the need for a unified classification system: a scoping review. Nutr Res Pract. 2025;19(3):331-344. doi:10.4162/nrp.2025.19.3.331 40496045 PMC12148626

