# Supplementary material for: Trends in Ultraprocessed Food Consumption Among Korean Children and Adolescents, 2007 to 2024
Source: JAMA Netw Open. 2026 Apr 7;9(4):e265528. doi: 10.1001/jamanetworkopen.2026.5528 (PMC13058760; doi:10.1001/jamanetworkopen.2026.5528)
Supplement: Supplement 1. — eTable 1. Korean NOVA Classification System Based on Product Characteristics eTable 2. Trends in Multivariable-Adjusted Percentage of Energy Intake From Nova Food Groups Among Korean Youths Aged 1 to 18 Years by KNHANES Cycle, 1998 to 2024 eTable 3. Trends in Multivariable-Adjusted Percentage of Energy Intake From Ultraprocessed Foods by Subcategories Among Korean Youths Aged 1 to 18 Years by KNHANES Cycle, 1998 to 2024 eFigure 1. Multivariable-Adjusted Changes in Percentage of Energy Intake From Subgroups of Ultraprocessed Food Consumption Among Korean Youths Aged 1 to 18 Years by KNHANES Cycle, 1998 to 2024 eFigure 2. Trends in Multivariable-Adjusted Percentage of Energy Intake From Ultraprocessed Foods Among Population Subgroups of Korean Youths Aged 1 to 18 Years by KNHANES Cycle, 1998 to 2024 [file jamanetwopen-e265528-s001.pdf]

Supplemental Online Content

Jung S, Lee EHL, Kim JY, Park S, Lee JE; the Ultra-Processed Food Working Group. Trends in ultraprocessed food consumption among Korean children and adolescents, 2007 to 2024. *JAMA Netw Open*. 2026;9(4):e265528. doi:10.1001/jamanetworkopen.2026.5528

eTable 1. Korean NOVA Classification System Based on Product Characteristics
eTable 2. Trends in Multivariable-Adjusted Percentage of Energy Intake From Nova Food Groups Among Korean Youths Aged 1 to 18 Years by KNHANES Cycle, 1998 to 2024
eTable 3. Trends in Multivariable-Adjusted Percentage of Energy Intake From Ultraprocessed Foods by Subcategories Among Korean Youths Aged 1 to 18 Years by KNHANES Cycle, 1998 to 2024
eFigure 1. Multivariable-Adjusted Changes in Percentage of Energy Intake From Subgroups of Ultraprocessed Food Consumption Among Korean Youths Aged 1 to 18 Years by KNHANES Cycle, 1998 to 2024
eFigure 2. Trends in Multivariable-Adjusted Percentage of Energy Intake From Ultraprocessed Foods Among Population Subgroups of Korean Youths Aged 1 to 18 Years by KNHANES Cycle, 1998 to 2024

This supplemental material has been provided by the authors to give readers additional information about their work.

© 2026 Jung S et al. *JAMA Network Open*.

eTable 1. Korean NOVA Classification System Based on Product Characteristics

Food Group	Food species	Food type	NOVA classification	Korean NOVA classification	Examples
Confectionery, bread or rice cakes	Confectionery	Confectionery	Group IV	Group III or Group IV	- Group III: Popcorns<br>- Group IV: Candy, chewing gum etc.
Beverages	Tea (leaching)	Leached tea, liquid tea, solid tea	Group IV	Group I or Group IV	- Group I: Simple extract tea<br>- Group IV: Contains food additives such as swelling agents and flavor enhancers
	Coffee			Group I or Group II or Group IV	- Group I: Drip coffee, espresso<br>- Group II: Brewed coffee<br>- Group IV: Mix & beverage
Korean fermented sauce	Soybean paste *(doenjang)*	Korean *meju*, improved *meju,* Korean *doenjang, doenjang*, etc.	Group IV	Group III or Group IV	- Group III: Traditional sauce<br>- Group IV: Mass production (raw material defatted soybean)
	Soy sauce *(ganjang)*	Korean *ganjang*, brewed *ganjang,*acid-decomposed *ganjang*, etc.		Group III or Group IV	- Group III: Traditional sauce<br>- Group IV: Mass production (acid hydrolysis)
	Gochujang	*Gochujang*		Group III or Group IV	- Group III: Traditional sauce<br>- Group IV: Mass production (raw material ingredients)
Seasoned food	Spice products	Natural spices		Group I or Group IV	- Group I: Dried raw material<br>- Group IV: Mixing several raw materials in liquid or powder form
Agricultural processed foods	Peanut or nut processed products	Peanut butter, peanut or nut processed products	Group IV	Group I or Group III or Group IV	- Group I: Peanut, almond, walnut<br>- Group III: Seasoned peanut and nuts<br>- Group IV: Peanut butter
	Other agricultural products	Processed fruits and vegetables, processed grains, processed beans, etc.		Group I or Group III or Group IV	Consider raw materials<br>- Group III:<br>· Possible to distinguish the raw materials<br>· Ingredients: Raw materials > industrial materials<br>- Group IV:

© 2026 Jung S et al. *JAMA Network Open*.

Food Group	Food species	Food type	NOVA classification	Korean NOVA classification	Examples
					· Impossible to distinguish the raw materials<br>· Ingredients: Raw materials > industrial materials
Processed meat products and packaged meat	Hams	Ham, raw ham, press ham		Group III or Group IV	- Group III: Traditional ham (ex. prosciutto, salami, jamon)<br>- Group IV: Processed ham
	Seasoned meat	Seasoned meat, ground processed meat products, processed ribs, etc.		Group III or Group IV	- Group III: Seasoned meat, processed ribs<br>- Group IV: Ground processed meat
	Meat-containing processed products		Group IV	Group III	
Processed egg products	Egg products	Whole egg liquid, egg yolk liquid, egg white liquid, whole egg flour, pidan, etc.		Group III or Group IV	Consider raw materials<br>- Group III:<br>· Possible to distinguish the raw materials<br>· Ingredients: Raw materials > industrial materials<br>- Group IV:<br>· Impossible to distinguish the raw materials<br>· Ingredients: Raw materials > industrial materials
Milk products	Fermented milk	Fermented milk, thick Fermented milk, fermented butter milk		Group I or Group III	- Group I: Fermented milk<br>- Group III: Fortified milk
	Butters	Butter, processed butter, butter oil		Group III or Group IV	- Group III:<br>· Ingredients: Raw materials > industrial materials<br>- Group IV:<br>· Ingredients: Raw materials > industrial materials
	Cheeses	Natural cheese, processed cheese	Group IV	Group III or Group IV	- Group III: Natural cheese<br>- Group IV: Processed cheese
Processed marine products	Seasoned laver			Group I or Group III	- Group I: Laver<br>- Group III: Seasoned laver

© 2026 Jung S et al. *JAMA Network Open*.

Food Group	Food species	Food type	NOVA classification	Korean NOVA classification	Examples
	Other processed marine products			Group I or Group III or Group IV	Consider raw materials<br>- Group III:<br>· possible to distinguish the raw material<br>· Ingredients: Raw materials > industrial materials<br>- Group IV:<br>· impossible to distinguish the raw material<br>· Ingredients: Raw materials > industrial materials
Animal processed foods	Insect processed foods			Group I or Group III	Consider raw materials
Honey and pollen processed foods	Pollen processed foods	Processed pollen		Group I	
Instant food	Instant·convenience food	Instant food, fresh-cut, ready to eat food	Group IV	Group III or Group IV	- Group III:<br>· possible to distinguish the raw material<br>· Ingredients: Raw materials > industrial materials<br>- Group IV:<br>· impossible to distinguish the raw material<br>· Ingredients: Raw materials > industrial materials

© 2026 Jung S et al. *JAMA Network Open*.

eTable 2. Trends in Multivariable-Adjusted Percentage of Energy Intake From Nova Food Groups Among Korean Youths Aged 1 to 18 Years by KNHANES Cycle, 1998 to 2024

	Weighted mean (SE)[a]										
	1998 (n=1298)	2001 (n=719)	2005 (n=555)	2007–2009 (n=5424)	2010–2012 (n=5014)	2013–2015 (n=4194)	2016–2018 (n=4053)	2019–2021 (n=3048)	2022–2024 (n=2785)	P for linear trend	2022-2024 vs. 1998, mean difference (SE)
Total energy intake (kcal)	1861.8 (33.5)	1723.5 (34.4)	1867.2 (39.6)	1645.5 (13.3)	1832.8 (14.5)	1840.0 (13.5)	1796.6 (14.3)	1713.0 (15.6)	1697.4 (14.2)	0.02	-26.1 (37.4)
**% energy intake from Nova groups**											
Unprocessed of minimally processed foods	69.2 (0.6)	62.1 (0.9)	64.2 (1.0)	64.5 (0.5)	60.8 (0.4)	56.0 (0.4)	53.7 (0.4)	52.7 (0.5)	51.5 (0.4)	<.001	-10.6 (1.0)
Processed culinary ingredients	3.2 (0.1)	3.4 (0.1)	4.1 (0.2)	3.9 (0.1)	3.8 (0.1)	4.1 (0.1)	4.0 (0.1)	4.3 (0.1)	4.7 (0.1)	<.001	1.3 (0.2)
Processed foods	7.0 (0.3)	10.6 (0.5)	7.4 (0.4)	6.7 (0.2)	6.8 (0.2)	8.3 (0.2)	9.6 (0.2)	10.2 (0.3)	10.5 (0.3)	<.001	-0.1 (0.6)
Ultra-processed foods	20.7 (0.6)	23.9 (0.9)	24.2 (1.1)	24.9 (0.5)	28.5 (0.4)	31.4 (0.4)	32.5 (0.4)	32.7 (0.5)	33.3 (0.5)	<.001	9.3 (1.0)

Abbreviations: KNHANES, Korea National Health and Nutrition Examination Survey.

[a] Estimated mean and SE were obtained using weighted linear regression model after adjusting for age, sex, residential area, and monthly household income.

[b] P values for linear trends were assessed by modeling the survey cycle as a continuous variable in survey-weighted linear regression models.

© 2026 Jung S et al. *JAMA Network Open*.

eTable 3. Trends in Multivariable-Adjusted Percentage of Energy Intake From Ultraprocessed Foods by Subcategories Among Korean Youths Aged 1 to 18 Years by KNHANES Cycle, 1998 to 2024

	Weighted mean (standard error)[a]										
	1998 (n=1298)	2001 (n=719)	2005 (n=555)	2007–2009 (n=5424)	2010–2012 (n=5014)	2013–2015 (n=4194)	2016–2018 (n=4053)	2019–2021 (n=3048)	2022–2024 (n=2785)	P for linear trend	2022-2024 vs. 1998, mean difference (SE)
**Total ultra-processed foods consumption (%kcal)**	20.7 (0.6)	23.9 (0.9)	24.2 (1.1)	24.9 (0.5)	28.5 (0.4)	31.4 (0.4)	32.5 (0.4)	32.7 (0.5)	33.3 (0.5)	<.001	9.3 (1.0)
**Subcategory**											0.8 (0.2)
**Industrial grain foods**	8.8 (0.4)	10.3 (0.7)	9.9 (0.8)	8.4 (0.3)	8.7 (0.3)	9.0 (0.3)	9.1 (0.2)	8.6 (0.3)	9.1 (0.3)	0.95	-1.2 (0.8)
Breads, rolls, and tortillas	0.8 (0.1)	0.6 (0.1)	0.8 (0.2)	1.1 (0.1)	1.2 (0.1)	1.3 (0.1)	1.1 (0.1)	1.2 (0.1)	1.6 (0.1)	<.001	0.9 (0.2)
Breakfast cereals	0.3 (0.04)	0.3 (0.06)	0.4 (0.09)	0.5 (0.04)	0.6 (0.05)	0.5 (0.04)	0.6 (0.05)	0.7 (0.06)	0.7 (0.06)	<.001	0.4 (0.1)
Muffins and quick breads	2.2 (0.2)	2.9 (0.4)	1.4 (0.2)	1.7 (0.1)	2.2 (0.1)	2.3 (0.1)	2.1 (0.1)	1.8 (0.1)	2.0 (0.1)	0.20	-1.0 (0.4)
Instant noodles	5.2 (0.4)	6.2 (0.7)	6.9 (0.7)	4.9 (0.2)	4.4 (0.2)	4.7 (0.2)	5.1 (0.2)	4.7 (0.2)	4.6 (0.2)	0.06	-1.6 (0.7)
Rice cakes	0.4 (0.1)	0.2 (0.03)	0.4 (0.2)	0.3 (0.05)	0.3 (0.1)	0.2 (0.04)	0.2 (0.03)	0.2 (0.03)	0.2 (0.04)	0.03	0.1 (0.05)
**Sweet snacks and sweets**	7.0 (0.4)	5.8 (0.4)	6.9 (0.5)	7 (0.2)	8.4 (0.2)	9.1 (0.2)	9.0 (0.2)	8.6 (0.2)	8.8 (0.3)	<.001	2.9 (0.5)
Snack	4.0 (0.3)	3.4 (0.4)	3.5 (0.4)	2.7 (0.1)	3.4 (0.1)	3.5 (0.1)	3.5 (0.1)	3.3 (0.1)	3.5 (0.2)	0.36	0.1 (0.4)
Cake	1.3 (0.2)	1.0 (0.2)	1.0 (0.2)	1.6 (0.1)	1.5 (0.1)	1.6 (0.1)	1.6 (0.1)	1.2 (0.1)	1.7 (0.1)	0.11	0.7 (0.3)
Candy, chocolate, caramel	0.7 (0.1)	0.5 (0.1)	0.4 (0.1)	0.6 (0.1)	1.2 (0.1)	1.4 (0.1)	1.6 (0.1)	1.6 (0.1)	1.7 (0.1)	<.001	1.2 (0.1)
Ice cream, desserts	1.0 (0.1)	1.0 (0.2)	2.0 (0.2)	2.2 (0.1)	2.4 (0.1)	2.6 (0.1)	2.3 (0.1)	2.5 (0.1)	1.9 (0.1)	<.001	1.0 (0.2)
**Processed meats including fish and poultry products**	1.4 (0.2)	2.1 (0.3)	1.7 (0.2)	2.6 (0.1)	2.9 (0.1)	3.8 (0.1)	4.5 (0.2)	5.7 (0.2)	5.9 (0.2)	<.001	3.8 (0.4)
Processed red meats	0.8 (0.1)	1.3 (0.2)	0.9 (0.2)	1.5 (0.1)	1.5 (0.1)	1.8 (0.1)	1.9 (0.1)	2.8 (0.1)	2.9 (0.1)	<.001	1.6 (0.2)
Fish and poultry products	0.6 (0.1)	0.8 (0.2)	0.8 (0.2)	1.1 (0.1)	1.4 (0.1)	2.0 (0.1)	2.6 (0.1)	2.9 (0.2)	3.0 (0.2)	<.001	2.2 (0.3)
**Flavored dairy foods and diary substitutes**	1.5 (0.1)	1.8 (0.1)	2 (0.1)	2.3 (0.1)	2.9 (0.1)	2.8 (0.1)	2.9 (0.1)	2.7 (0.1)	3.1 (0.1)	<.001	1.3 (0.2)
Flavored milk	0.2 (0.05)	0.3 (0.1)	0.5 (0.1)	0.7 (0.1)	0.7 (0.1)	0.7 (0.1)	0.8 (0.1)	0.8 (0.1)	1.0 (0.1)	<.001	0.7 (0.1)
Flavored yogurts	0.9 (0.1)	1.0 (0.1)	1.0 (0.1)	1.0 (0.1)	1.4 (0.1)	1.4 (0.1)	1.6 (0.1)	1.4 (0.1)	1.6 (0.1)	<.001	0.6 (0.1)
Dairy drinks and dairy	0.4 (0.04)	0.5 (0.1)	0.5 (0.1)	0.6 (0.1)	0.8 (0.1)	0.6 (0.1)	0.5 (0.04)	0.5 (0.1)	0.5 (0.04)	0.04	0.03 (0.1)

© 2026 Jung S et al. *JAMA Network Open*.

substitutes											
**Sugar-sweetened beverages**	0.6 (0.1)	1.0 (0.1)	1.5 (0.2)	1.8 (0.1)	2.8 (0.1)	3.4 (0.1)	3.2 (0.1)	3.0 (0.1)	3.2 (0.1)	<.001	2.2 (0.2)
Soft drinks	0.2 (0.1)	0.7 (0.1)	0.6 (0.2)	0.7 (0.1)	1.0 (0.1)	1.3 (0.1)	1.4 (0.1)	1.4 (0.1)	1.1 (0.1)	<.001	0.5 (0.1)
Fruit and other sweetened drinks	0.5 (0.1)	0.4 (0.1)	0.8 (0.1)	1.0 (0.1)	1.7 (0.1)	2.1 (0.1)	1.8 (0.1)	1.6 (0.1)	2.1 (0.1)	<.001	1.7 (0.1)
**Other**	0.3 (0.1)	1.3 (0.1)	1.6 (0.1)	1.7 (0.1)	1.8 (0.1)	1.9 (0.1)	2.4 (0.1)	2.8 (0.1)	3.0 (0.1)	<.001	1.7 (0.1)
Fats, condiments, and sauces	0.1 (0.1)	1.1 (0.1)	1.4 (0.1)	1.3 (0.1)	1.5 (0)	1.8 (0.1)	2.3 (0.1)	2.6 (0.1)	2.8 (0.1)	<.001	1.7 (0.1)
Other ultra-processed foods (baby formula)	0.2 (0.03)	0.2 (0.03)	0.2 (0.03)	0.4 (0.05)	0.3 (0.05)	0.1 (0.03)	0.1 (0.02)	0.2 (0.04)	0.2 (0.04)	0.001	-0.03 (0.04)
**Ready-to-heat and -eat mixed dishes**	1.1 (0.2)	1.7 (0.4)	0.6 (0.2)	1.1 (0.1)	1.1 (0.1)	1.4 (0.1)	1.3 (0.1)	1.3 (0.1)	0.3 (0.04)	0.02	-1.4 (0.4)
Pizza, hamburgers, sandwiches	0.8 (0.2)	1.5 (0.4)	0.3 (0.2)	0.4 (0.1)	0.5 (0.1)	1.0 (0.1)	1.1 (0.1)	1.1 (0.1)	0.01 (0.02)	0.50	-1.5 (0.4)
Instant soups, curries	0.3 (0.1)	0.2 (0.1)	0.3 (0.1)	0.6 (0.1)	0.5 (0.1)	0.4 (0.04)	0.3 (0.02)	0.3 (0.03)	0.3 (0.03)	<.001	0.1 (0.1)

Abbreviations: KNHANES, Korea National Health and Nutrition Examination Survey.

[a] Estimated mean and SE were obtained using weighted linear regression model after adjusting for age, sex, residential area, and monthly household income.

[b] P values for linear trends were assessed by modeling the survey cycle as a continuous variable in survey-weighted linear regression models.

© 2026 Jung S et al. *JAMA Network Open*.

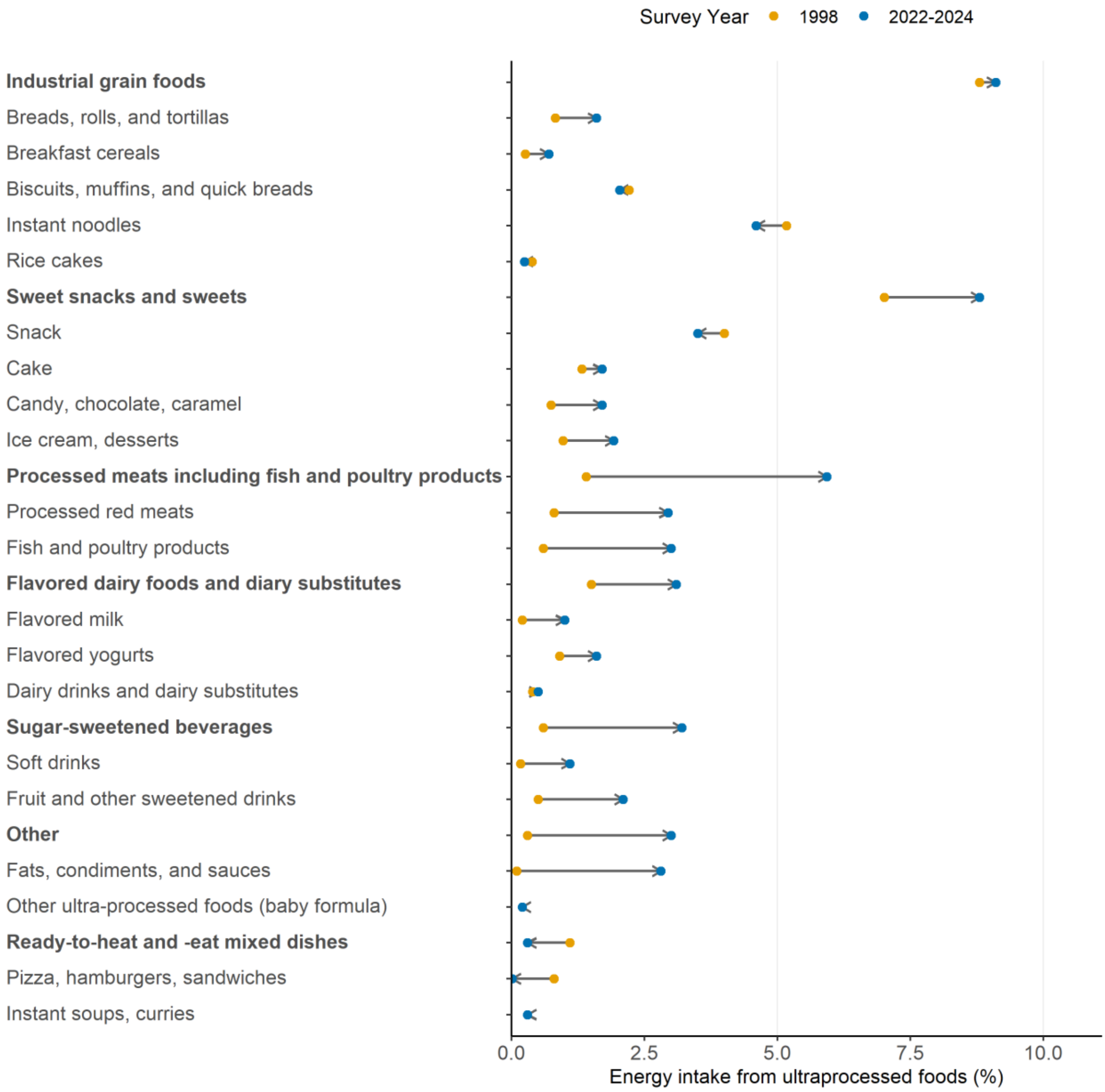

eFigure 1. Multivariable-Adjusted Changes in Percentage of Energy Intake From Subgroups of Ultraprocessed Food Consumption Among Korean Youths Aged 1 to 18 Years by KNHANES 9 cycle, 1998 to 2024

Abbreviation: KNHANES, Korea National Health and Nutrition Examination Survey.
Note: Estimated mean and SE were obtained using weighted linear regression model after adjusting for age, sex, residential area, and monthly household income.

© 2026 Jung S et al. *JAMA Network Open*.

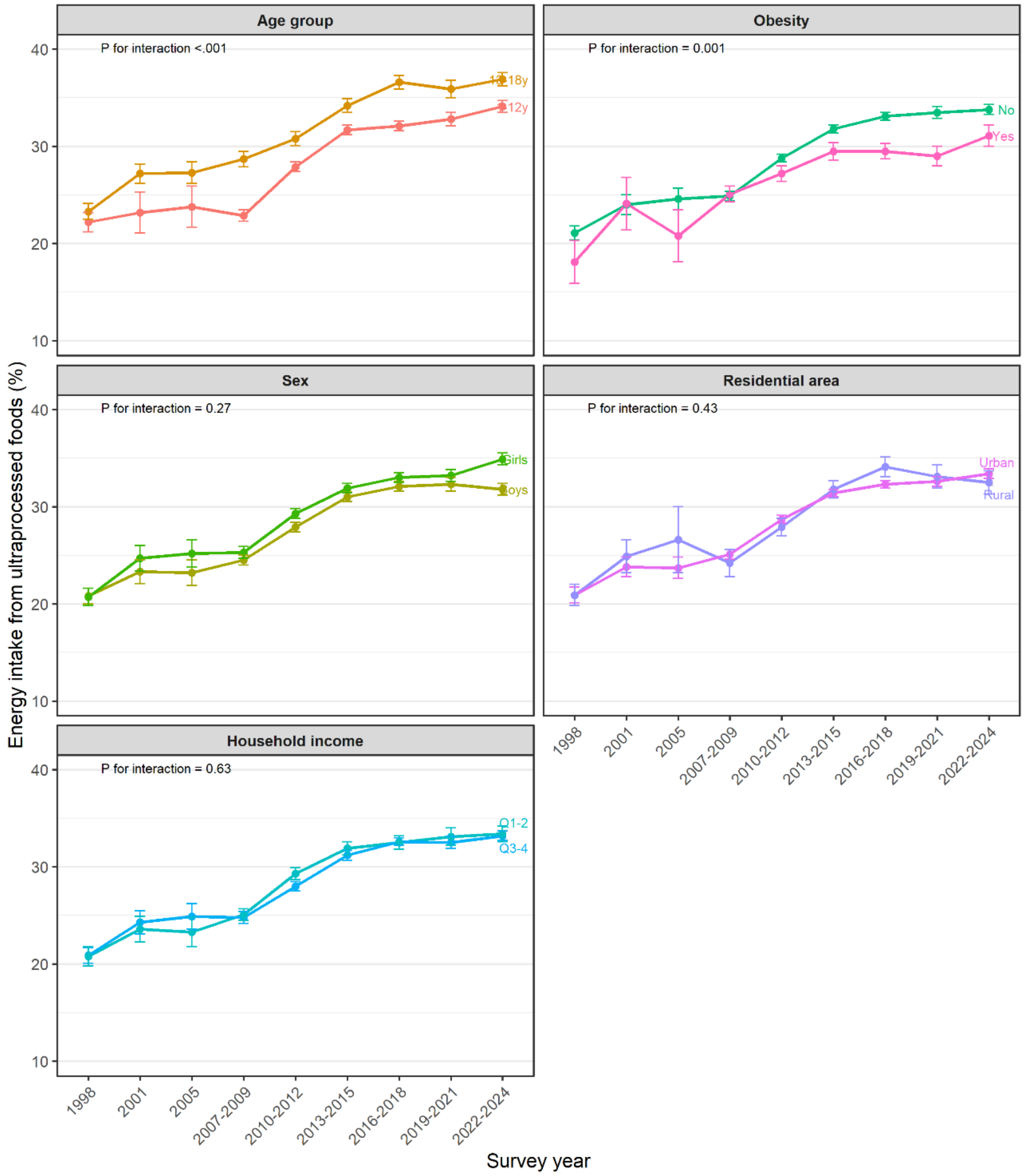

eFigure 2. Trends in multivariable-adjusted percentage of energy intake from ultra-processed foods among population subgroups of Korean youths aged 1 to 18 years by KNHANES cycle, 1998–2024

Abbreviation: KNHANES, Korea National Health and Nutrition Examination Survey.
Note: Estimated mean and SE were obtained using weighted linear regression model after adjusting for age, sex, residential area, and monthly household income except the corresponding subgroup variables.

© 2026 Jung S et al. *JAMA Network Open*.
