# Supplementary material for: Trends in Ultraprocessed Food Consumption Among Korean Children and Adolescents, 2007 to 2024
Source: JAMA Netw Open. 2026 Apr 7;9(4):e265528. doi: 10.1001/jamanetworkopen.2026.5528 (PMC13058760; doi:10.1001/jamanetworkopen.2026.5528)
Supplement: Supplement 2. — Data Sharing Statement [file jamanetwopen-e265528-s002.pdf]

Data Sharing Statement

Jung. Trends in Ultraprocessed Food Consumption Among Korean Children and Adolescents, 2007 to 2024. *JAMA Netw Open*. Published April 07, 2026.
doi:10.1001/jamanetworkopen.2026.5528

Data
Data available: Yes
Data types: Deidentified participant data
How to access data: https://knhanes.kdca.go.kr/knhanes/main.do.
When available: With publication

Supporting Documents
Document types: None

Additional Information
Who can access the data: All data are publicly available at, https://knhanes.kdca.go.kr/knhanes/main.do.
Types of analyses: All data are publicly available at, https://knhanes.kdca.go.kr/knhanes/main.do.
Mechanisms of data availability: All data are publicly available at, https://knhanes.kdca.go.kr/knhanes/main.do.
